# TWEAK Activates the Non-Canonical NFκB Pathway in Murine Renal Tubular Cells: Modulation of CCL21

**DOI:** 10.1371/journal.pone.0008955

**Published:** 2010-01-29

**Authors:** Ana B. Sanz, Maria D. Sanchez-Niño, Maria C. Izquierdo, Aniela Jakubowski, Pilar Justo, Luis M. Blanco-Colio, Marta Ruiz-Ortega, Rafael Selgas, Jesús Egido, Alberto Ortiz

**Affiliations:** 1 Fundación Jiménez Díaz, Universidad Autónoma de Madrid, Fundación Renal Iñigo Alvarez de Toledo, Madrid, Spain; 2 Department of Immunobiology, Biogen Idec, Inc., Cambridge, Massachusetts, United States of America; 3 Servicio de Nefrologia, Fundación para la Investigación Biomédica del Hospital Universitario La Paz, Madrid, Spain; Institut Pasteur, France

## Abstract

TWEAK is a member of the TNF superfamily of cytokines that contribute to kidney tubulointerstitial injury. It has previously been reported that TWEAK induces transient nuclear translocation of RelA and expression of RelA-dependent cytokines in renal tubular cells. Additionally, TWEAK induced long-lasting NFκB activation suggestive of engagement of the non-canonical NFκB pathway. We now explore TWEAK-induced activation of NFκB2 and RelB, as well as expression of CCL21, a T-cell chemotactic factor, in cultured murine tubular epithelial cells and in healthy kidneys in vivo. In cultured tubular cells, TWEAK and TNFα activated different DNA-binding NFκB complexes. TWEAK-induced sustained NFκB activation was associated with NFκB2 p100 processing to p52 via proteasome and nuclear translocation and DNA-binding of p52 and RelB. TWEAK, but not TNFα used as control), induced a delayed increase in CCL21a mRNA (3.5±1.22-fold over control) and CCL21 protein (2.5±0.8-fold over control), which was prevented by inhibition of the proteasome, or siRNA targeting of NIK or RelB, but not by RelA inhibition with parthenolide. A second NFκB2-dependent chemokine, CCL19, was upregulates by TWEAK, but not by TNFα. However, both cytokines promoted chemokine RANTES expression (3-fold mRNA at 24 h). In vivo, TWEAK induced nuclear NFκB2 and RelB translocation and CCL21a mRNA (1.5±0.3-fold over control) and CCL21 protein (1.6±0.5-fold over control) expression in normal kidney. Increased tubular nuclear RelB and tubular CCL21 expression in acute kidney injury were decreased by neutralization (2±0.9 vs 1.3±0.6-fold over healthy control) or deficiency of TWEAK (2±0.9 vs 0.8±0.6-fold over healthy control). Moreover, anti-TWEAK treatment prevented the recruitment of T cells to the kidney in this model (4.1±1.4 vs 1.8±1-fold over healthy control). Our results thus identify TWEAK as a regulator of non-canonical NFκB activation and CCL21 expression in tubular cells thus promoting lymphocyte recruitment to the kidney during acute injury.

## Introduction

Acute kidney injury (AKI) and progressive loss of renal function are associated with interstitial inflammation and tubular injury [1]. Infiltration by leukocytes depends on the local expression of inflammatory cytokines and chemokines. Tubular epithelial cells release an array of cytokines in response to various immune and non-immune factors, contributing to attraction of inflammatory cells to the kidney [2], [3]. Members of the TNF superfamily regulate several cell responses, including proliferation, differentiation, cell death and inflammation [4]. Some of these cytokines, such as TNF and FasL, have been extensively studied in kidney diseases and shown to be involved in renal damage [5]–[8]. More recently, Tumor necrosis factor-like weak inducer of apoptosis (TWEAK, TNFSF12) has been implicated in glomerular and tubulointerstitial inflammatory responses [9]–[13], cell death in the presence of additional inflammatory mediators, [10], [11] and cell proliferation in the absence of such mediators [14]–[17]. TWEAK mediates its biologic activitities by signaling via its receptor Fibroblast growth factor-inducible 14 (Fn14) [9], [11]. It was previously shown that TWEAK-induced chemokine secretion in tubular cells was mediated by the RelA NFκB subunit [9]. Additionally, a sustained NFκB activation of unknown significance was noted, consistent with the NFκB non-canonical pathway activation [9]. In this pathway, the NFκB-inducing kinase activity (NIK) is required for the phosphorylation/ubiquitination and proteasomal processing of the IκB protein NFκB2 p100 to NFκB2 p52 [18]. NFκB2 p52/RelB dimer translocates to the nucleus and activates transcription of specific gene targets [19]. Only a few cytokines are able to engage this pathway, including B-cell activating factor (BAFF) [20], [21], CD40 ligand [22], and receptor activator of NF-kappa-B ligand (RANKL) [23], but not TNF [18]. Non-canonical activation of NFκB2 leads to transcription of a set of genes different from those regulated by canonical NFκB activation [19]. Following lymphotoxin (LT)-β receptor (LTβR) ligation in splenocytes NFκB2 targets include CC chemokine ligand 21/secondary lymphoid chemokine (CCL21)/(SLC), EBI-1-ligand chemokine (ELC/CCL19), B lymphocyte chemoattractant (BLC/CXCL13), stromal cell-derived factor-1 α (SDF-1-α/CXCL12) and BAFF [19]. However, the regulation and targets of the non-canonical pathway in renal cells are poorly understood. TWEAK was reported to activate NFκB2 in fibroblasts, but the functional consequences were not studied and whether this pathway is active in epithelial and, specifically, in renal epithelial cells, is unknown [24]. Different cell types activate NFκB in a different manner when exposed to the same stimulus [25]. CCL21 is T-cell chemotactic factor that has been recently related to renal tubulointerstitial injury [26]. However the factors that contribute to CCL21 upregulation in renal tubulointerstitial injury are poorly characterized.

We now report that TWEAK activates NFκB2 and RelB and induces the expression of CCL21a mRNA and CCL21 protein in cultured murine tubular epithelial cells and in healthy kidneys and that TWEAK antagonism reduces tubular CCL21 and lymphocyte T infiltration in folic acid-induced AKI. To our knowledge this is the first report of non-canonical NFκB pathway regulators and targets in tubular cell injury.

## Methods

### Cells and Reagents

MCT murine proximal tubular epithelial cells were cultured in RPMI 1640, (GIBCO, Grand Island, NY, USA), 10% decomplemented fetal bovine serum, 2 mM glutamine, 100 U/mL penicilin and 10 µg/mL streptomycin, in 5% CO_2_ at 37°C.[9]. Recombinant human soluble TWEAK (Alexis, Läufelfingen, Switzerland) was used at 100 ng/ml and murine TNFα (Immugenex. Los Angeles, CA) at 30 ng/mL based on prior TWEAK and TNFα dose-response induction of cytokine production and cell death induction in MCT cells [9], [27]. The NFκB inhibitor parthenolide (Sigma, St. Louis, MO) was used at 10 µM, the proteasome inhibitor MG132 (Calbiochem, La Jolla, CA) at 20 µM, the proteasome inhibitor lactacystin (Sigma) at 10 µm.

### Western Blot

Samples of tissue and cells were homogenized in lysis buffer were separated by 10–12% SDS-PAGE under reducing conditions [9]. Primary antibodies were rabbit polyclonal anti-p100/52 (1∶500, a gift of Helen Cooper), rabbit polyclonal anti-RelB (1∶500, Santa Cruz Biotechnology, CA), rabbit polyclonal anti IκBα (1∶500, Santa Cruz Biotechnology), rabbit polyclonal anti IκBβ (1∶500, Santa Cruz Biotechnology), rabbit polyclonal anti phospho-IΚΚα (1∶500, Santa Cruz Biotechnology), rabbit polyclonal anti-CCL21 (1∶500, Santa Cruz Biotechnology). Blots were then probed with anti-α-tubulin (1∶2000, Sigma) for total proteins and anti-lamin B (1∶500, Santa Cruz Biotechnology) for nuclear proteins and levels of expression were corrected for minor differences in loading.

### Quantitative Reverse Transcription-Polymerase Chain Reaction

One µg RNA isolated by Trizol (Invitrogen, UK) was reverse transcribed with High Capacity cDNA Archive Kit and real-time PCR was performed on a ABI Prism 7500 PCR system (Applied Biosystems, Foster City, CA) using the DeltaDelta Ct method. Expression levels are given as ratios to GAPDH. Pre-developed primer and probe assays were from Applied [9].

### Electrophoretic Mobility Shift Assay (EMSA)

Cells were resuspended in buffer A (10 mmol/L HEPES, pH 7.8, 15 mmol/L KCl, 2 mmol/L MgCl2, 1 mmol/L PMSF, 0.1 mmol/L EDTA, and 1 mmol/L DDT) and homogenized. Nuclei and cytosolic fractions were separated by centrifugation at 1000×g 10 min. Nuclei (pellet) were washed twice in buffer A and resuspended in the same buffer, with a final concentration of 0.39 mol/L KCl. Nuclei were extracted for 1 h at 4°C and centrifuged at 100,000×g 30 min. Supernatants containing nuclei were dialyzed in buffer C (50 mmol/L HEPES, pH 7.8, 50 mmol/L KCl, 10% glycerol, 1 mmol/L PMSF, 0.1 mmol/L EDTA, and 1 mmol/L DDT) and then cleared by centrifugation and stored at −80°C. The protein concentration was determined by the bicinchoninic acid method. EMSA was carried out as previously described [9]. For supershift assays, nuclear extracts were incubated with 1 µg anti-RelA, anti-RelB, or anti-p52 antibody, all from Santa Cruz Biotechnology, for 1 h at 37°C before incubation with the labeled probe.

### ELISA-Based NFκB Assay

Cells were stimulated with 100 ng/mL TWEAK for different time periods. In some cases cells were pre-treated with parthenolide or lactacystin 1 hour before TWEAK addition. RelA, RelB and p52 subunits in nuclear extract were measured by their binding to an oligonucleotide containing the NFκB consensus site using TransAM NFκB Family Kit (Active Motif, Carlsbad, CA).

### siRNA Transfection

Cells were seeded at a 3×10^5^ in 6-wells plates and transfected on the following day with 20 nM siRNA oligonucleotides by using lipofectamine reagent (Invitrogen). After 72 h, the transfected cells were treated with 100 ng/mL TWEAK for 24 h and harvested for Western blot analysis. siRNA were synthesized by Santa Cruz Biotechnology.

### Confocal Microscopy

Cells plated onto Labtek™ slides were fixed in 4% paraformaldehyde for 10 min and ermeabilized in 0.2% Triton X-100/PBS for additional 10 min, washed in PBS and incubated with rabbit polyclonal anti-RelB (1∶75, Santa Cruz Biotechnology), rabbit polyclonal anti-p100/p52 (1∶50, a gift of Helen Cooper) followed by FITC secondary antibody (1∶200, Sigma). Nuclei were counterstained with propidium iodide. Cells were analysed by confocal microscopy.

### Gene Expression Arrays

One µg RNA isolated by Trizol (Invitrogen UK) was reverse transcribed with High Capacity cDNA Archive Kit (Applied Biosystems). The resulting cDNA (0.5 ng) was added to the 2x Universal PCR Master Mix (Applied Biosystems). Aliquots of the mixture were placed into each well of a card with 384 wells. This product has 96 TaqMan® Gene Expression Assays loaded in singletons into the array's 384 wells, that include primer sets for 92 mouse genes known to have implications in immune response plus four housekeeping genes (TaqMan® Mouse Immune Array, Applied Biosystems). Real-time PCR was performed with a 7900HT Fast Real-Time PCR System (Applied Biosystems). Finally, data were analyzed based on the ΔΔCt method with normalization of the raw data to the four housekeeping genes.

### Animal Model

Studies were conducted in accordance with the NIH Guide for the Care and Use of Laboratory Animals. C57/BL6 mice (12- to 14-week-old)(IFFA-CREDO, Barcelona, Spain) were treated i.p. with 0.75 µg/mouse of TWEAK or saline vehicle (200 µl 0.9% NaCl). Mice were killed 4 h (n = 6) or 24 h (n = 7) after injection. The dose of TWEAK was calculated based on cell culture experiments, when TWEAK exhibited optimal activity at 10–100 ng/mL, assuming an extracellular volume of 7.5 mL/mouse and was previously shown to induce biological responses in kidneys [9].

Folic acid nephropathy is a classical model of AKI [9], [28]–[30]. C57/BL6 mice (12- to 14-week-old) received a single i.p. injection of folic acid (Sigma) 250 mg/kg in 0.3 M sodium bicarbonate or vehicle and mice were killed 24 h later [9]. Mice were dosed ip with either 200 µg neutralizing anti-TWEAK mAb (clone P2D10, Biogen Idec) [9] or 200 µg isotype IgG (mouseIgG2a, clone P1.17, Biogen Idec) (n = 6 per group). Animals received the mAb treatments one-day prior to the folic acid injection. In a second set of experiment, TWEAK knock out (KO) (Biogen Idec)[11] mice on the C57Bl/6 background strain received i.p. folic acid injection and they were killed 24 h later. The kidneys were perfused in situ with cold saline before removal. One kidney was snap-frozen in liquid nitrogen for RNA and protein studies and the other fixed and paraffin embedded.

### Immunohistochemistry

Immunohistochemistry was carried out as previously described on paraffin-embedded 5 µm tissue sections [9]. Primary antibodies were rabbit polyclonal anti-RelB (1∶50, Santa Cruz), rabbit polyclonal anti-p100/p52 (1∶20, Santa Cruz), goat polyclonal anti-CCL21 (1∶90, R&D Systems, Minneapolis, MN). Sections were counterstained with Carazzi's hematoxylin. Negative controls included incubation with a non-specific immunoglobulin of the same isotype as the primary antibody. CCL21 staining was evaluated by a quantitative scoring system using Image Pro Plus Software (Media cybernetics, Bethesda, MD) in 10 randomly chosen fields (20x) per kidney [9]. Samples were examined in a blinded manner.

CD3 cells were stained with rabbit polyclonal anti-CD3 (1∶300, Dako Diagnostics, Barcelona, Spain), using Dako REAL™ EnVision™ kit (Dako). The total number of CD3 positive lymphocytes was quantitated in 20 randomly chosen fields (x40) using Image-Pro Plus software. Samples were examined in a blinded manner.

### Statistics

Statistical analysis was performed using SPSS 11.0 statistical software. Results are expressed as mean ± SEM. Significance at the p<0.05 level was assessed by Student's t test for two groups of data and ANOVA for three of more groups.

## Results

### TWEAK Induces NFκB2 Processing, p52/RelB Nuclear Translocation and DNA-Binding in Renal Tubular Epithelial Cells

**Figure 1:**
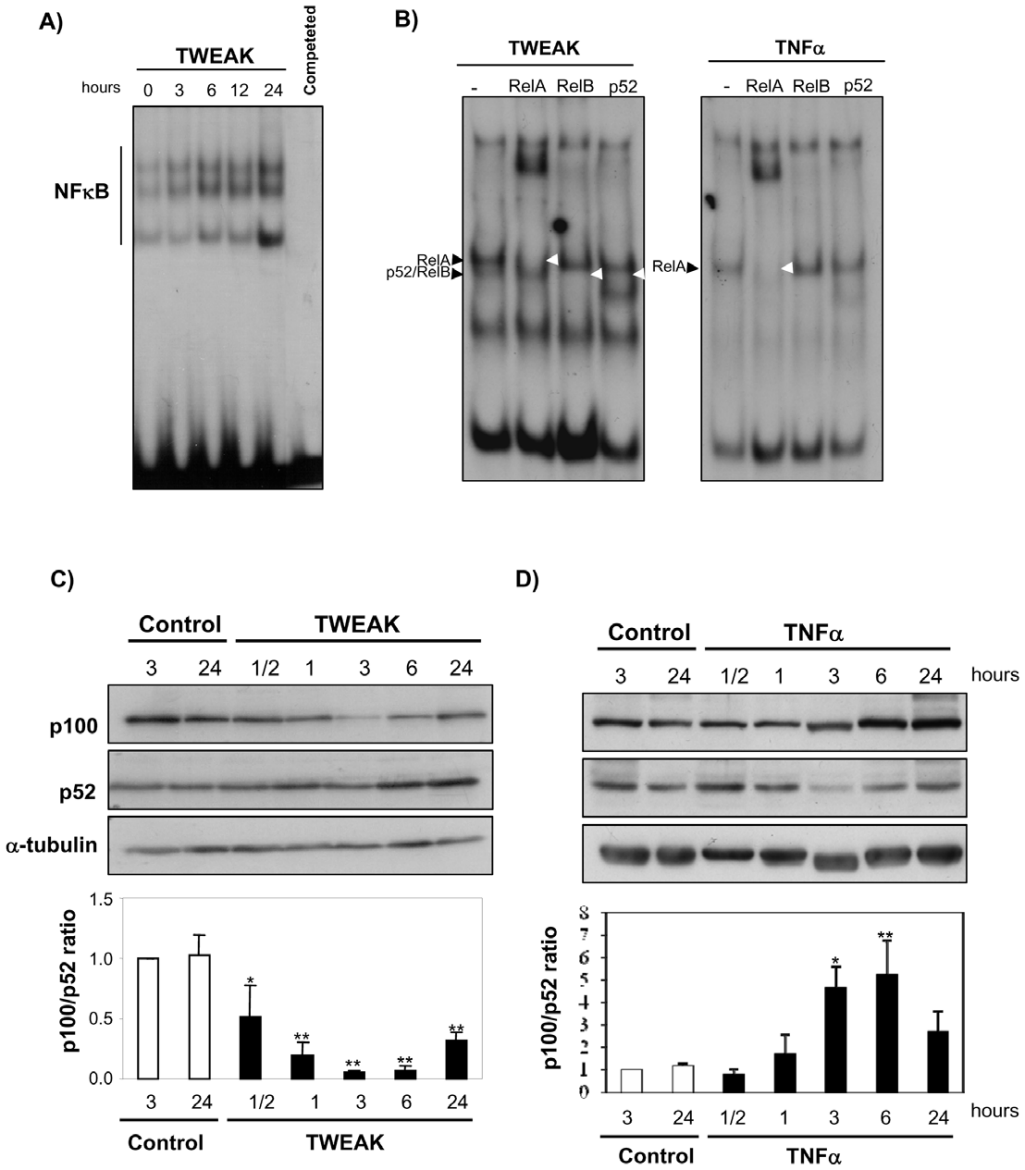
Different DNA-binding NFκB complexes induced by TWEAK and TNFα in renal tubular cells. **A**) Time-course of NFκB activation in MCT cells treated with 100 ng/mL TWEAK for indicated time periods. Representative EMSA of four independent experiments. **B**) Supershift analysis of nuclear extracts from tubular cells treated with TWEAK or TNFα for 24 h. Bands were further separated by the use of a 3% acrylamide gel. Complex 1 consists of two bands (black arrowheads), one containing RelA, which is shared by both cytokines, and another containing RelB and p52, present only in TWEAK-stimulated cells. Bands disappearing in the presence of antibodies marked by empty arrowheads. Representative EMSA of three independent experiments. **C,D**) Tubular cell lysates were analyzed by Western blot probed with anti-p100/p52. (**C**) TWEAK time-response. Mean ± SEM of four independent experiments. *p<0.001 vs control; **p<0.0001 vs control. (**D**) TNFα time-response. Mean ± SEM of three independent experiments. *p<0.02 vs control; **p<0.008 vs control.

We had previously described a delayed NFκB activation induced by TWEAK in tubular cells (**Figure 1A**) [9]. Supershift assays demonstrated the presence of RelB and p52 in TWEAK-induced DNA binding NFκB complexes, while RelA was present in both TNFα- and TWEAK-induced complexes (**Figure 1B**). The RelA and RelB/p52 containing bands migrated very close, similar to results reported in HT29 cells following LTβR ligation [19]. The addition of the p52 antibody resulted in the appearance of an even faster migrating complex. This faster migration has been previously been described in HT29 cells [31]. These results indicate that TWEAK induces non-canonical, sustained NFκB activation in tubular cells. On the other hand, in whole cell protein extracts TWEAK decreased NFκB2 p100 and increased the amount of its degradation product p52 in a time-dependent manner (**Figure 1C**). This was not observed with TNFα (**Figure 1D**). In fact, TNFα induces the expression of p100. These results suggest that TWEAK, but not TNFα, could activate NFκB2.

**Figure 2:**
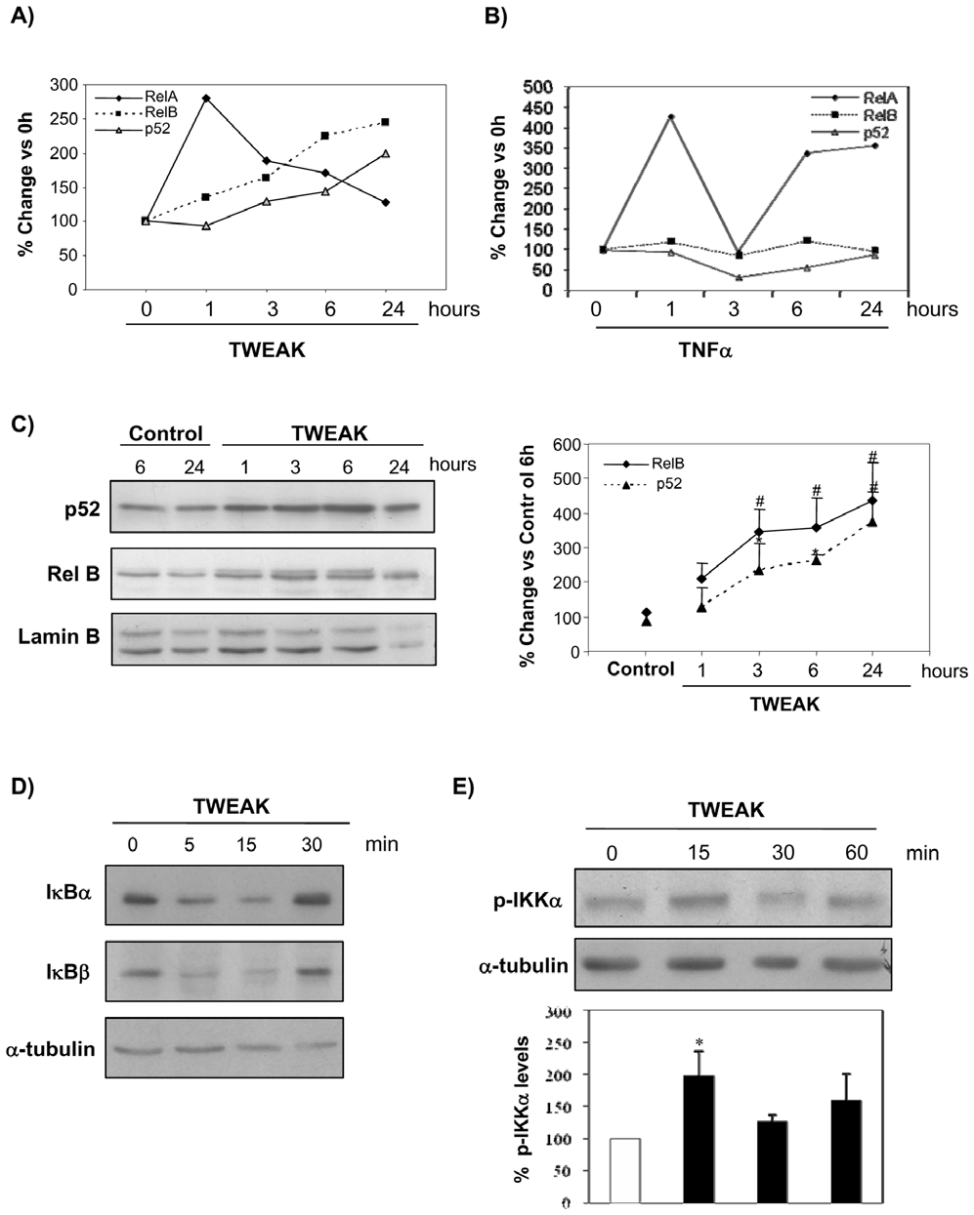
Nuclear translocation of p52 and RelB in TWEAK-stimulated tubular cells. **A, B**) Temporal pattern of nuclear translocation of RelA, RelB and p52 in tubular cells stimulated with TWEAK, (**A**), or TNFα (**B**). ELISA of nuclear proteins binding to an oligonucleotide containing the NFκB consensus site. **C**) Tubular cells were stimulated with 100 ng/mL TWEAK and nuclear cell lysates obtained at various time points were analyzed by Western blot for p52 and RelB. Representative Western blot and densitometric quantification. Mean ± SEM of four independent experiments. *p<0.04 vs control; #p<0.009 vs control. No significant differences were observed between controls at different time points and they were grouped. **D**) TWEAK induces degradation of the inhibitory subunits IkBα and IkBβ, in a time-course consistent with canonical NFκB pathway activation. Representative western blot of three independent experiments. **E**) TWEAK induces phosporylation of IKKα subunit of IKK complex. Representative Western blot and quantification of three independent experiments. Mean ± SEM of three experiments. *p<0.05 vs control.

**Figure 3:**
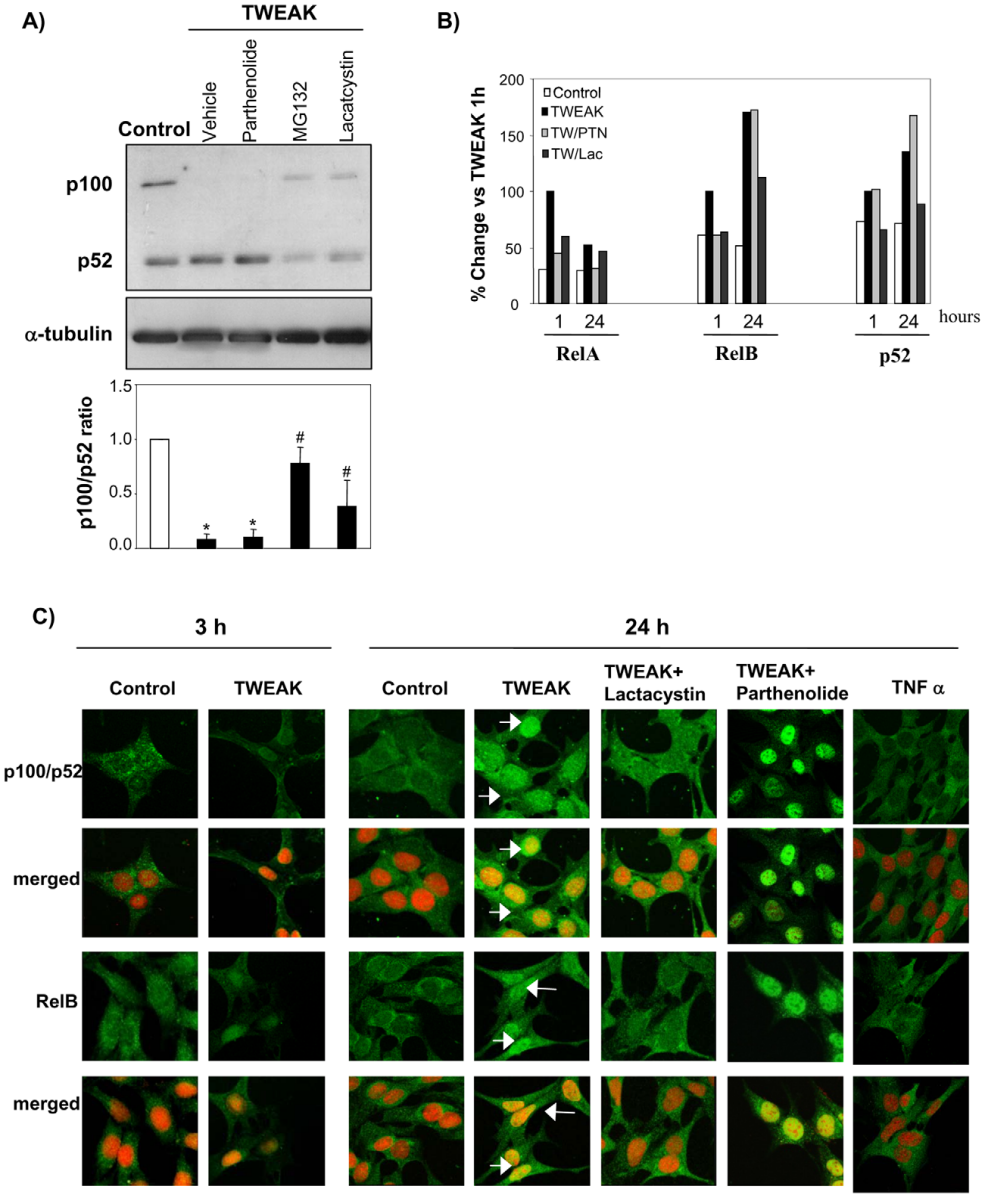
Proteasome activity are required for TWEAK-induced p100 processing. **A**) Tubular cells pre-treated with 10 µM parthenolide, 10 µM lactacystin or 20 µM MG132 were stimulated with 100 ng/ml TWEAK for 6 h. Mean ± SEM of four experiments. *p<0.0001 vs control; #p<0.02 vs TWEAK alone. Representative Western blots and quantification. **B**) Treatment with parthenolide does not prevent the nuclear translocation of RelB or p52, whereas lactacystin prevents this nuclear translocation. ELISA of nuclear proteins binding to an oligonucleotide containing the NFκB consensus site. **C**) Confocal microscopy. TWEAK induced nuclear translocation of RelB and p52 (both in green) at 3 h and 24 h that was prevented by lactacystin but not by parthenolide. TNFα does not induce RelB and p52 (both in green) nuclear translocation at 24 h. Propidium iodide is shown in orange. Note nuclear yellowish color in merged images when the proteins translocate to the nucleus (arrows). Representative pictures of three independent experiments.

TWEAK reportedly induced early and transient nuclear translocation of RelA in tubular cells [9]. We now show that TWEAK induces a progressive time-dependent increment in nuclear p52 and RelB, as assessed by ELISA of nuclear extracts (**Figure 2A**) Western blot (**Figure 2C**), and confocal microscopy (**Figure 3C**). An ELISA of nuclear NFκB subunits confirmed the early and transient nature of the increase in nuclear RelA and the progressive increase in nuclear RelB and p52 in MCT cells treated with TWEAK (**Figure 2A**), whereas TNFα only induces nuclear RelA translocation (**Figure 2B**). In MCT cells TWEAK induces degradation of IκBα and IκBβ (**Figure 2D**), and this was temporarily associated with translocation of the RelA subunit of NFκB from the cytoplasm to the nucleus [9]. The activation of the IKKα subunit of the IKK complex is necessary for NFκB2 activation via the non-canonical pathway [18]. In this regard, we observed by western blot that TWEAK induces IKKα phosphorylation (**Figure 2E**).

### Proteasome Activity Mediates TWEAK-Induced Non-Canonical NFkB2 Activation

The proteasome inhibitors, MG132 and lactacystin reduced the processing of p100 to p52 induced by TWEAK **(** **Figure 3A**). However, parthenolide, a sesquiterpeno lactone that inhibits RelA activation by preventing the degradation of IκB-α [9], [32] did not prevent p100 processing (**Figure 3A**). Parthenolide inhibits nuclear translocation of RelA, as previously observed by confocal microscopy and Western blot [9] while not modulating the nuclear translocation of RelB and p52 as assessed by ELISA of nuclear extract and confocal microscopy (**Figure 3B,C**). However, the proteasome inhibitor lactacystin inhibited nuclear translocation of RelB and p52, as assessed by ELISA of nuclear extracts and confocal microscopy (**Figure 3B,C** **)**.

### TWEAK-Induced NFκB2 Activation Increases CCL21a Expression in Renal Tubular Cells

**Figure 4:**
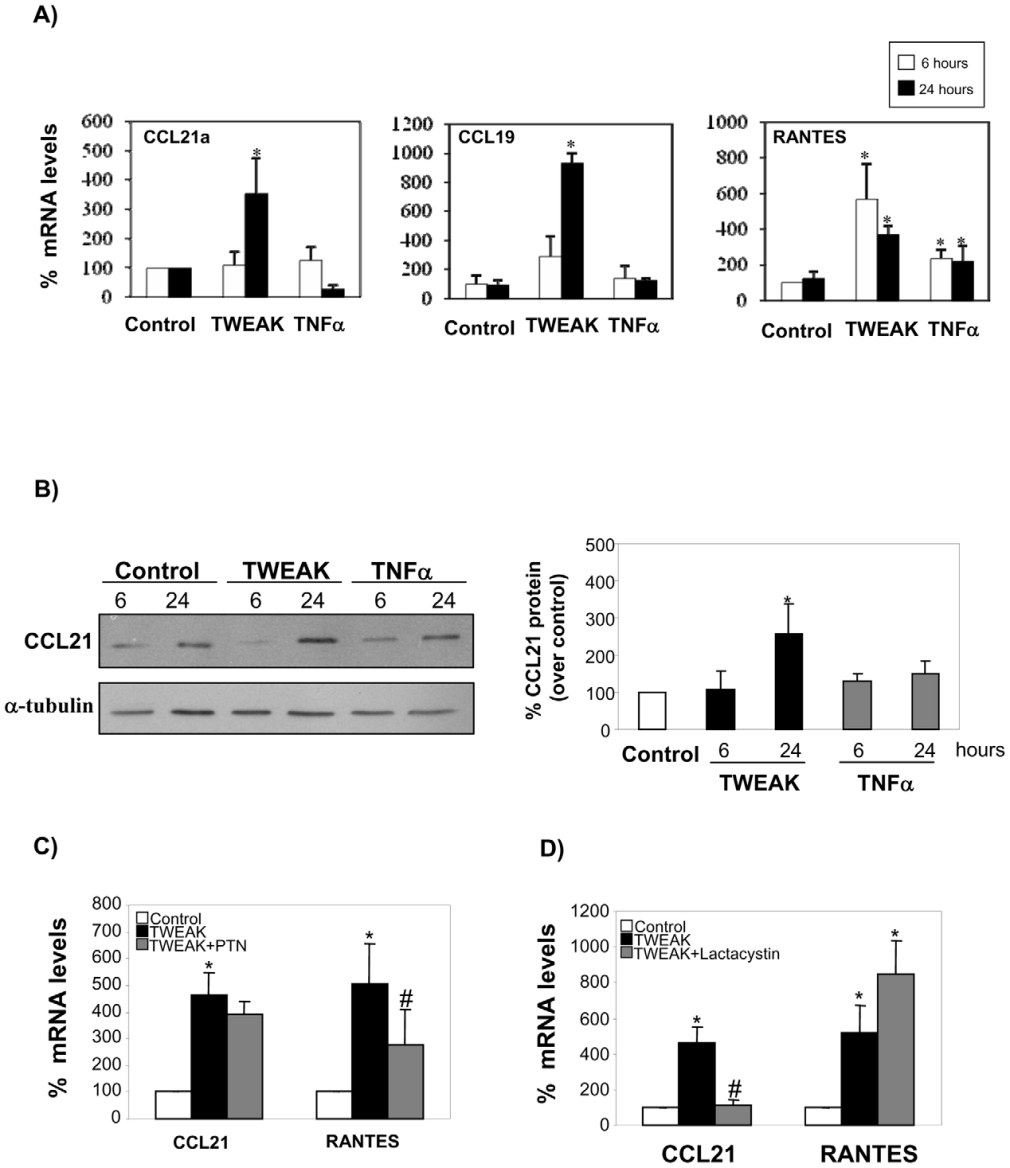
TWEAK induces the expression of NFκB2 targets, CCL21 and CCL19, in tubular cells. **A**) Time-dependent increase in CCL21a, CCL19 and RANTES mRNA expression in tubular cells treated with 100 ng/mL TWEAK or 30 ng/ml TNFα. Real time RT-PCR. Mean ±SEM of four independent experiments. *p<0.04 vs control. **B**) Time-dependent increase in CCL21 protein expression in tubular cells treated with 100 ng/mL TWEAK or 30 ng/mL TNFα. Mean ±SEM of four independent experiments. *p<0.03 vs control. **C**) Parthenolide (PTN) does not prevent TWEAK-induced CCL21a mRNA up-regulation at 24 h. Real time RT-PCR. Mean ±SEM of four experiments. *p<0.04 vs control; #p<0.04 vs TWEAK alone. **D**) Lactacystin prevents TWEAK-induced CCL21a mRNA up-regulation at 24 h. Real time RT-PCR. Mean ±SEM of four experiments. *p<0.04 vs control; #p<0.03 vs TWEAK alone.

CCL21 was previously reported to be a gene target of the non-canonical NFκB2 pathway in splenocytes [19], [33]. We selected CCL21 as a potential target of NFκB2 activation in renal cells because it modulates T cell recruitment and CCL21 has recently been reported to play a role in tubulointerstitial injury [26]. Additionally, the pathways that regulate its expression in the kidney are unknown. As previously reported basal CCL21a mRNA levels were 40-fold higher in control spleen than in control kidney (not shown) [34]. TWEAK induced the expression of CCL21a mRNA in cultured tubular cells. The time-course of CCL21a stimulation paralleled that of RelB/p52 accumulation in nuclei, increasing up to 24 h (**Figure 4A**). We used RANTES as a TNF-inducible control. At the protein level, TWEAK, but not TNFα, induced the expression of the CCL21 cytokine (**Figure 4B**). Unsupervised hierarchical cluster analysis of an array of 94 immune response-related genes did not disclose differences between TNF and TWEAK treated cells at either 4 or 24 h: samples stimulated with either cytokine clustered together and could not be separated. However, induction of CCL19 mRNA, a known target of NFκB2 [19], [35], was only observed in TWEAK stimulated cells. Induction of CCL19 mRNA with a time-course consistent with the RelB/p52 accumulation in nuclei by TWEAK, but not by TNF, was then confirmed by qRT-PCR (**Figure 4A**).

Parthenolide inhibits the degradation of IκB-α and RelA nuclear translocation and thus, canonical NFκB activation [9], [32]. Parthenolide did not prevent the CCL21a up-regulation induced by TWEAK (**Figure 4C**) suggesting that RelA does not mediate its transcription. RANTES is a RelA target with a delayed pattern of mRNA expression induction by inflammatory mediators, due to a delayed access of RelA to its encoding DNA [36]. We had previously observed that TWEAK increases the expression of RANTES in a temporal pattern similar to CCL21, that is delayed with respect to the induction of other *bone fide* RelA targets such as MCP-1 [9], we now confirmed this observation (**Figure 4A**). Parthenolide prevented TWEAK induction of RANTES mRNA, as previously reported in detail [9], but did not modulate CCL21 expression (**Figure 4C**). By contrast, lactacystin prevented CCL21a, but not RANTES expression induced by TWEAK (**Figure 4D**).

**Figure 5:**
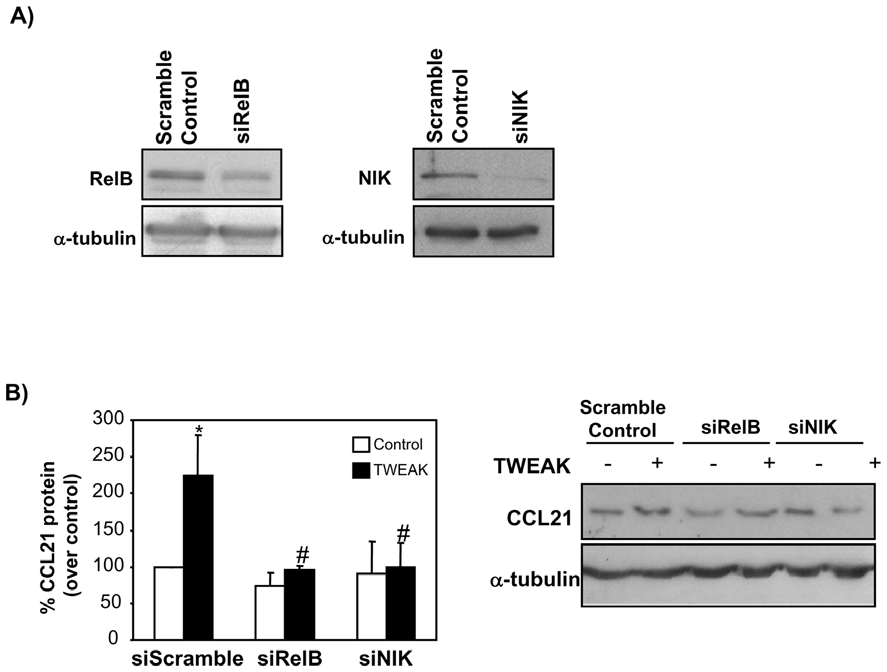
RelB and NIK down-regulation by siRNA reduces TWEAK-induced CCL21 expression in tubular cells. **A**) RelB and NIK protein levels are efficiently down-regulation by siRNA. Tubular cells were transfected with either a siRNA oligonucleotide targeting RelB, NIK or scrambled control. RelB and NIK expression was assessed 72 hours after transfection by Western blot. Representative Western blot of four independent experiments. **B**) Tubular cells transfected 72 h earlier were treated with TWEAK for 24 h. RelB or NIK siRNA targeting prevented CCL21 upregulation. Representative Western blot and quantification. Mean ± SEM of four independent experiments. *p<0.02 vs control. #p<0.02 vs scrambled Control + TWEAK.

The non-canonical NFκB pathway requires NFκB-inducing kinases (NIK) activation and the formation of RelB/p52 complexes [18]. In this regard, siRNA targeting of either RelB or NIK (**Figure 5A**) prevented CCL21 upregulation in cultured tubular cells (**Figure 5B**).

### TWEAK Modulates Renal CCL21a Expression In Vivo and T Lymphocyte Infiltration

**Figure 6:**
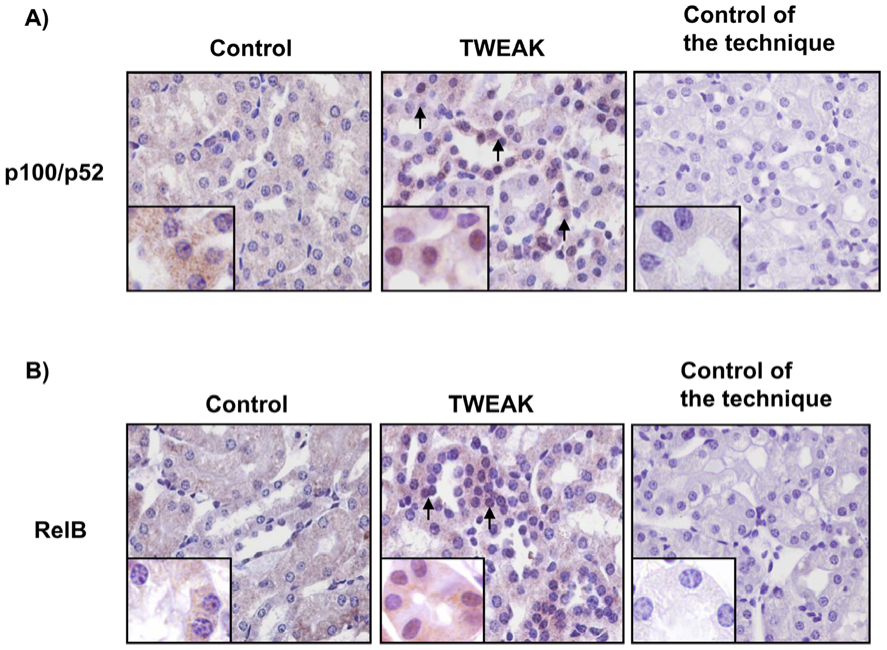
TWEAK induces nuclear translocation of p52 and RelB in vivo. **A**) p100/p52 immunohistochemistry 24 h following TWEAK or vehicle injection. Nuclear p52 is observed in renal tubules from mice treated with TWEAK (arrows). Original magnification ×400. Detail ×1000. Pictures representative of 6/7 animals per group. **B**) RelB immunohistochemistry 24 h following TWEAK or vehicle injection. Nuclear RelB is observed in tubules from mice treated with TWEAK (arrows). Original magnification ×400. Detail ×1000. Controls for the technique are stained with non-specific IgG. Nuclear staining is present in samples stained with specific anti-p100/52 and anti-RelB as compared with the isotype control. Pictures representative of 6/7 animals per group.

**Figure 7:**
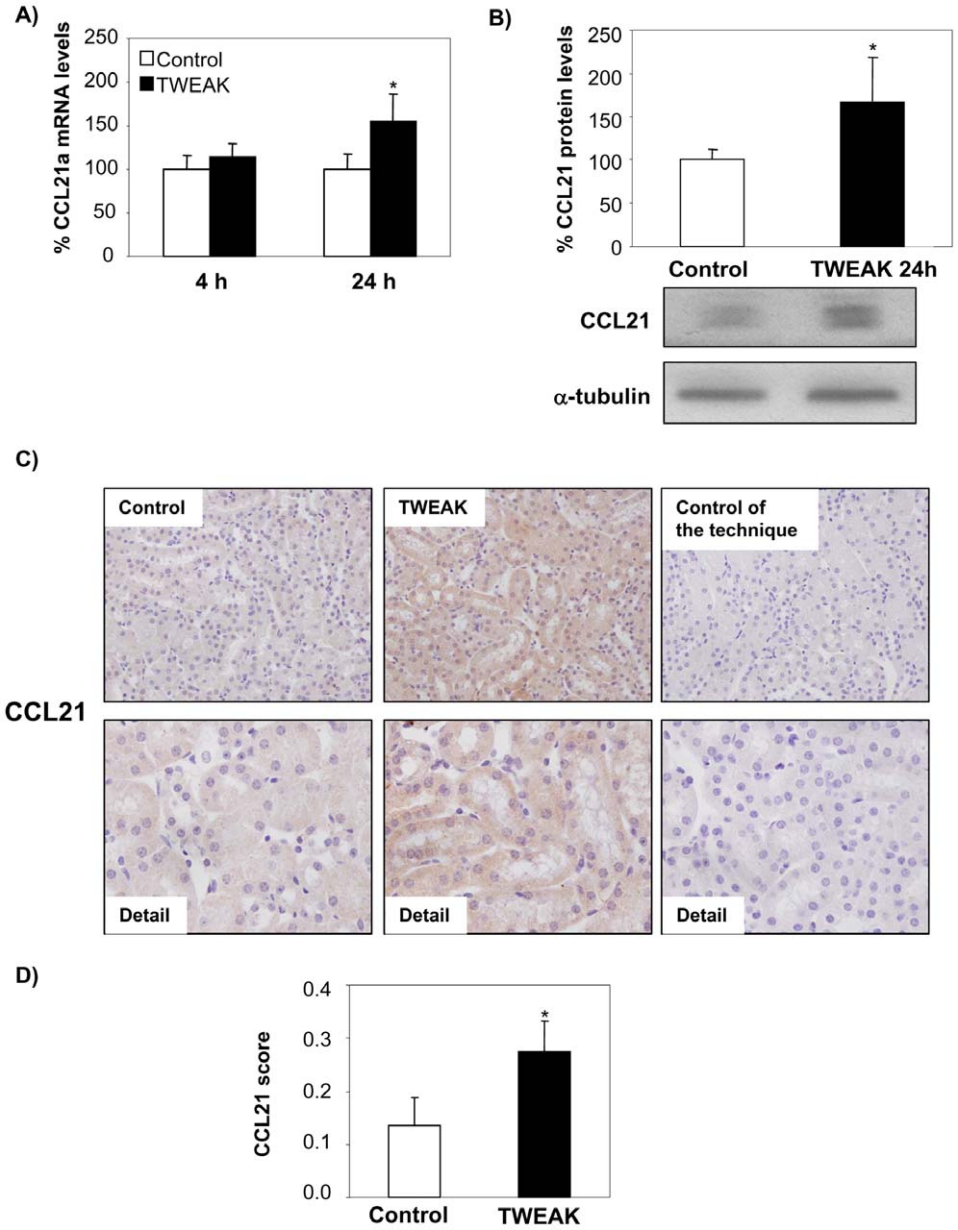
TWEAK modulates kidney CCL21 expression in vivo. **A**) TWEAK treatment of healthy mice increases kidney CCL21a mRNA at 24 h. RNA obtained from the whole kidney. Real time RT-PCR. Mean ±SEM of 6/7 animals per group. *p<0.05 vs control 24 h. **B**) Increased kidney CCL21 protein induced by TWEAK as assessed by Western blot. Mean ± SEM of 6/7 animals per group. *p<0.05 vs control 24 h. **C**) CCL21 protein localized to tubular epithelium of kidneys 24 h following TWEAK injection. Original magnification ×200. Detail ×400. Controls for the technique are stained with non-specific IgG. **D**) Quantification of CCL21 staining as mean ± SEM of 5 animals per group. *p<0.04 vs control.

In vivo TWEAK administration to healthy control mice promotes the nuclear translocation of RelB and p52 in tubular cells (**Figure 6**). Consistent with the cell culture data, TWEAK increases both CCL21a mRNA and protein expression in healthy kidneys at 24 h but not at 41 h (**Figure 7A,B**). Furthermore CCL21 protein was localized to tubular cells (**Figure 7C,D** **)** As previously published, TWEAK did not modify serum creatinine levels and only caused mild signs of tubular injury, but not cell death, when administered to healthy mice [9].

**Figure 8:**
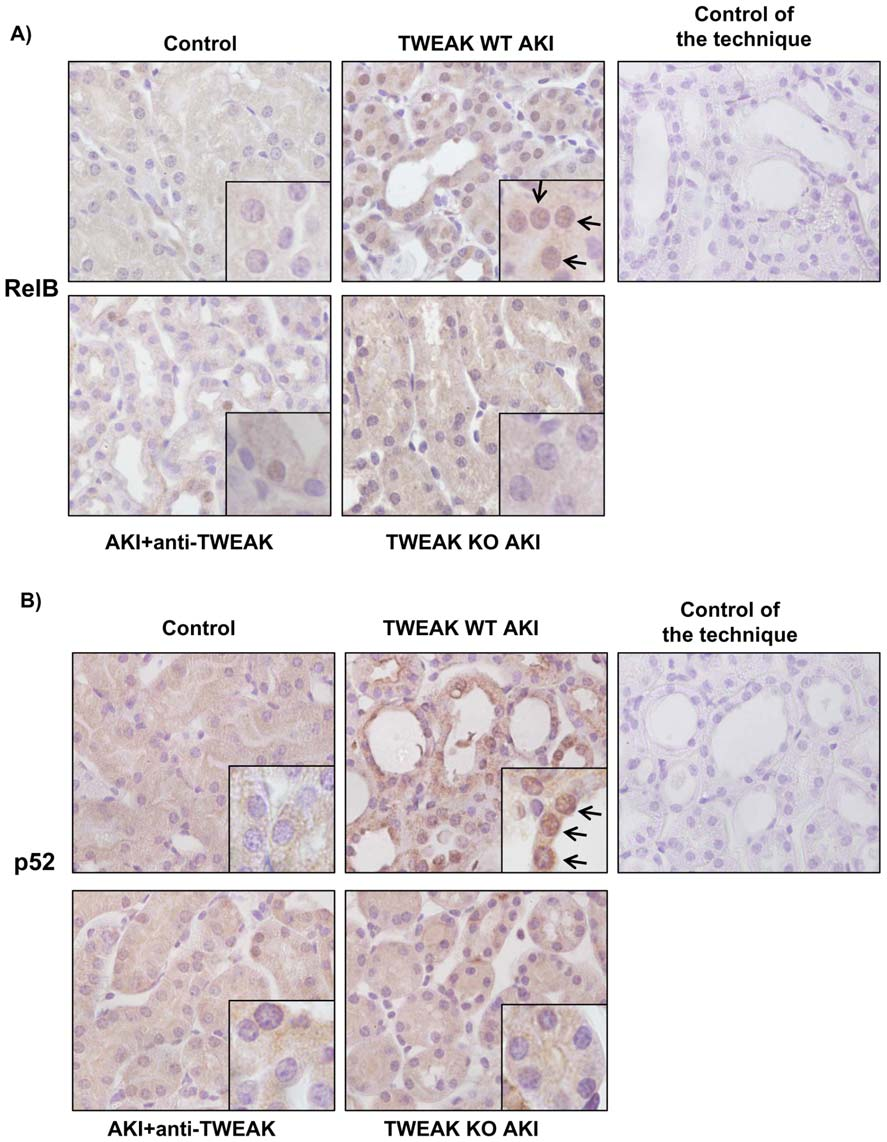
Absence or antagonism of TWEAK decreases RelB and p52 nuclear translocacion in AKI. AKI was induced by a single folic acid overdose. This model is characterized by increased renal tubular expression of TWEAK and Fn14 [9], [10]. **A**) RelB immunohistochemistry, **B**) p52 immunohistochemistry. Note nuclear cell localization of RelB and p52 in mice with acute kidney injury (AKI) at 24 h (arrows). TWEAK deficiency or antibody mediated inhibition decreases RelB and p52 positive nuclei. Controls for the technique are stained with non-specific IgG. Original magnification ×400. Detail ×1000. Pictures representative of 5 animals per group.

Folic acid-induced AKI shares many of the features of human AKI, including tubular cell death and proliferation, tubulointerstitial inflammation and delayed fibrosis [9], [28]–[30]. We had previously observed a key role for TWEAK in tubulointerstitial inflammation in this model, as TWEAK and Fn14 expression by tubular cells is increased (by 30% and 3-fold as assessed by Western blot at 24 h) and TWEAK targeting by anti-TWEAK antibodies decreased inflammation and preserved renal function [9], [10], [37]. We now report RelB and p52 nuclear translocation, whole kidney CCL21a mRNA upregulation and tubular cell expression of CCL21 protein 24 h following induction of AKI (**Figure 8** **,** **9**). Blockade of TWEAK by anti-TWEAK antibodies or absence of TWEAK in TWEAK−/− mice prevented the RelB and p52 nuclear translocation and the increased CCL21 expression in AKI (**Figure 8** **,** **9**). In vivo, CCL21 is chemotactic for T lymphocytes [33], [34], and recently, TWEAK was shown to induce kidney infiltration by T cells [38]. In this regard, we observed increased renal interstitial CD3 positive lymphocytes in AKI at 24 h, and neutralization of TWEAK and TWEAK absence decreased the number of CD3 lymphocyte (**Figure 10**).

**Figure 9:**
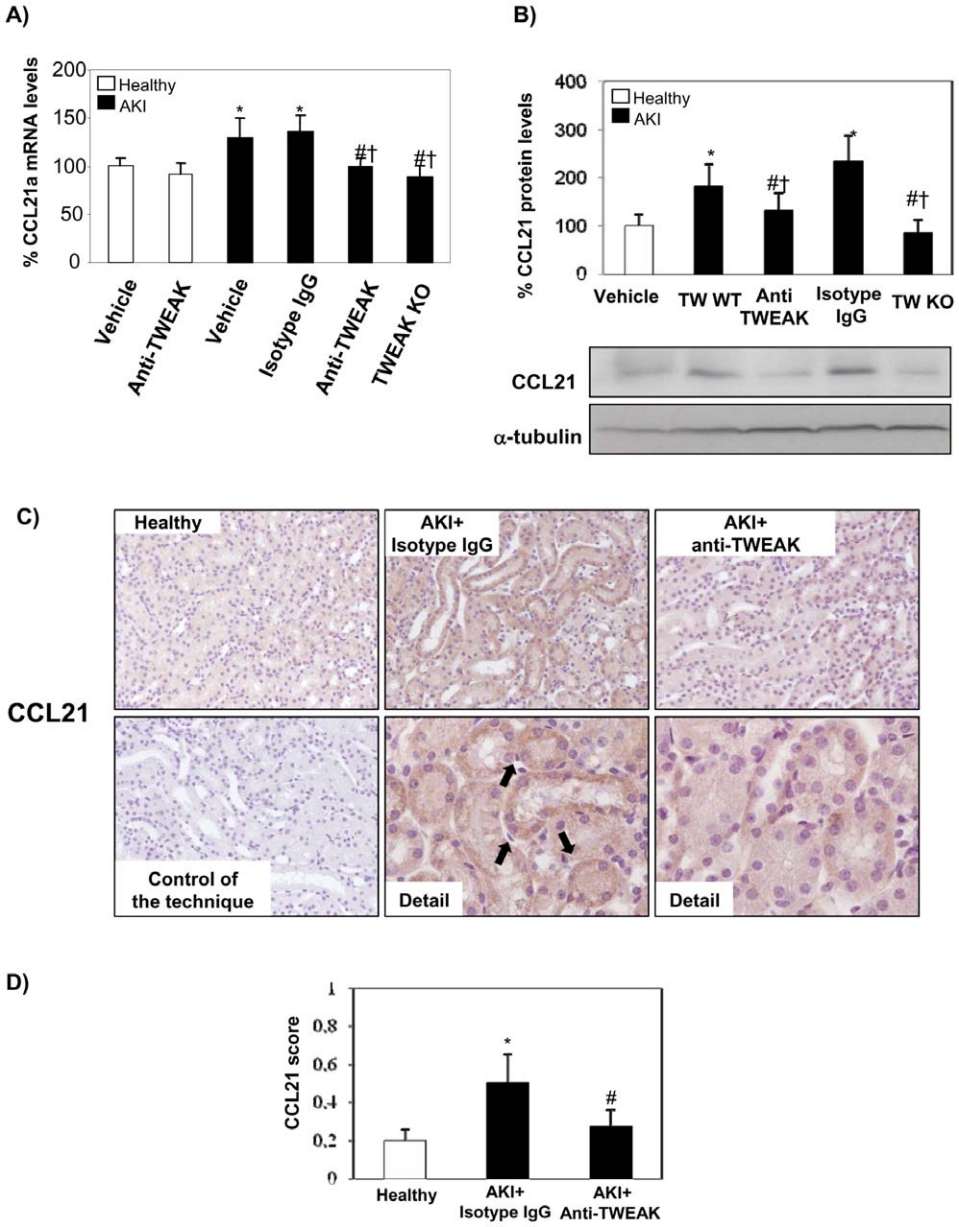
Absence or antagonism of TWEAK decreases CCL21 in AKI. **A**) TWEAK deficiency or anti-TWEAK antibodies decrease kidney CCL21a mRNA in AKI at 24 h following injury. Real time RT-PCR. Mean ±SEM of 6/7 animals per group. *p<0.02 vs control, #p<0.05 vs AKI, †p<0.05 vs AKI + isotype IgG. **B**) Total kidney CCL21 protein expression in AKI at 24 h following renal injury. Measured by Western blot. Mean ± SEM of 6/7 animals per group. *p<0.03 vs control, #p<0.05 vs AKI, †p<0.05 vs AKI + sotype IgG. Representative Western blot showing CCL21 protein expression in AKI model. **C**) CCL21 immunohistochemistry. Note epithelial cell localization of increased CCL21 expression in mice with AKI, when compared with healthy control or mice with AKI treated with anti-TWEAK. Original magnification ×200. Detail ×400. Controls for the technique are stained with non-specific IgG. **D**) Quantification of CCL21 as mean ± SEM of 6/7 animals per group. *p<0.004 vs control, #p<0.002 vs AKI.

**Figure 10:**
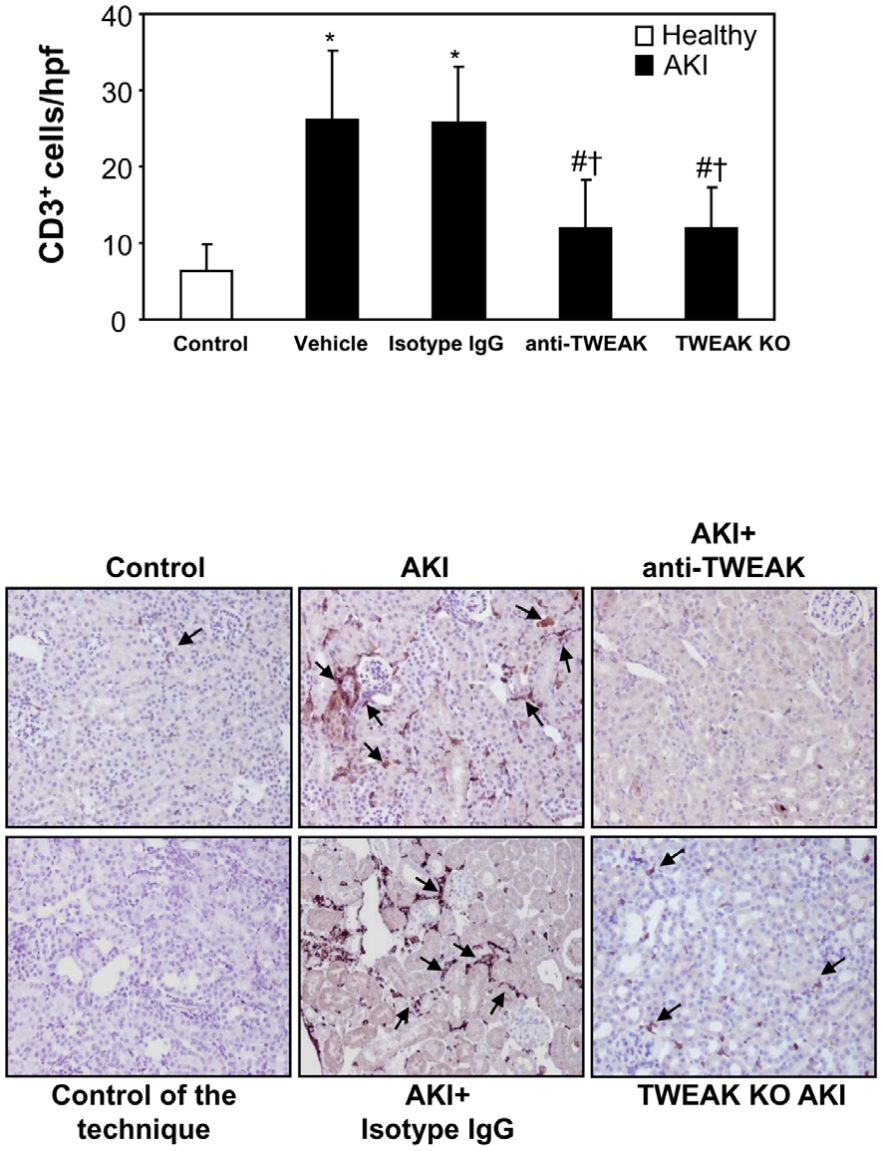
Antagonism or absence of TWEAK decreases CD3^+^ lymphocytes in AKI. The increased number of interstitial lymphocytes stained with anti-CD3 (arrows) in AKI kidneys was reduced by anti-TWEAK antibody treatment or in TWEAK KO mice. Original magnification ×200. Controls for the technique are stained with non-specific Ig. Quantification as mean ± SEM of 6/7 animals per group. *p<0.0001 vs control, #p<0.001 vs AKI, †p<0.002 vs AKI + Isotype IgG.

## Discussion

It was previously reported that TWEAK has a proinflammatory effect dependent on the canonical NFκB pathway in cultured tubular epithelial cells [9]. In these cells TWEAK induces the RelA-dependent synthesis of MCP-1, IL-6 and RANTES and, in addition, TWEAK induces sustained NFκB activation [9]. We now provide evidence that supports the notion that in tubular epithelium TWEAK induces the sequential activation of two separate signaling cascades for NFκB that promote the transcription of different sets of genes. Initially canonical activation of RelA promotes the release of MCP-1 and RANTES [9]. In tubular cells TWEAK also activates the non-canonical NFκB pathway requiring NIK, p100 processing and RelB and leading the synthesis of CCL21. Thus CCL21 is identified as a TWEAK-regulated gene in tubular cells in culture and in vivo. This information identifies a new signaling cascade in tubular cell injury and will help develop new targets for therapeutic intervention [9].

NFκB activates the transcription of different genes with specificity and kinetics that vary in a gene-, stimulus- and cell-specific manner. Delayed kinetics of gene transcription may be due to involvement of the non-canonical NFκB pathway or to decreased DNA accessibility [18], [36].

Delayed NFκB-dependent gene transcription may be the consequence of NFκB activation through the non-canonical pathway [18]. TWEAK was observed to induce non-canonical NFκB activation in fibroblasts [24]. However, no consequences of this activation were studied and it is unknown if the pathway is active in epithelial cells [24]. Indeed no targets of non-canonical NFκB by TWEAK have been previously characterized. There is more information on NFκB2 targets in lymphoid cells. Thus, LTbetaR ligation induces CCL21/SLC, BLC, ELC, SDF1 and BAFF expression in the spleen through the non-canonical pathway of NFκB activation [19]. The kinetics of RelA, RelB and p52 activation by LtbetaR ligation in spleen were similar to those observed with TWEAK treatment of tubular cells in the present paper [19]. Peripheral lymph node expression of CCL21 was down regulated in NFκB2−/− mice, suggesting the requirement for NFκB2 for its expression [12]. Of these potential NFκB2 targets we chose CCL21 for further study. CCL21 is a high affinity ligand for chemokine receptor 7 (CCR7). Activation of CCR7 is chemotactic for thymocytes, T cells, mature dendritic cells, and, to a lesser extent, B cells [39]. CCL21 plays a role in mediating homing of lymphocytes to secondary lymphoid organs. In addition it induces chemotaxis and proliferation in mesangial cells [39], [40]. Moreover, CCR7-positive fibrocytes migrate into the kidney in response to SLC/CCL21 and contribute to kidney fibrosis induced by unilateral ureteral obstruction in mice [26]. CCL21 also binds to the chemokine receptors CCR11 and CXCR3 [34]. While CCL21 expression by high endothelial venules (HEV)-like vessels was previously emphasized, most CCL21 was expressed outside these vessels [26]. CCL21 had been previously reported to be expressed by podocytes and endothelial lymphoid cells [40], [41]. However, its expression by tubular cells was poorly characterized and the factors that regulate renal CCL21 expression had not been identified.

In cultured tubular cells TWEAK induced a delayed expression of CCL21. This contrasts to the early peak (3 h) of MCP-1 and IL-6 gene expression which was abolished by the RelA inhibitor parthenolide [9]. In vivo TWEAK administration increased kidney CCL21a expression at 24 h, but not at 4 h. This is in contrast to the early (4 h) increase in RelA-dependent genes MCP-1 and Il-6 under the same experimental conditions [9]. The temporal pattern of CCL21 mRNA expression in vitro and in vivo was reminiscent of the delayed expression of RANTES in tubular cells. A delayed access of RelA to DNA has been implicated in the delayed induction of RANTES mRNA [36]. When access to DNA is delayed, sustained RelA availability that persists up to when DNA has become accessible for transcription is required [36]. RANTES induction by TWEAK shared with MCP-1 its response to the RelA inhibitor parthenolide [9]. We now show persistent low level RelA binding to DNA (up to 24 h) in tubular cells stimulated with TWEAK. Taken together these data are consistent with the hypothesis that the canonical NFκB pathway is responsible for the delayed RANTES upregulation in TWEAK-stimulated tubular cells. However, contrary to the findings with RANTES [9], the expression of CCL21 was not prevented by parthenolide. Functional studies indicate that the delayed induction of CCL21 is dependent on the delayed activation of the non-canonical NFκB pathway and requires NIK, NFκB2 processing and RelB. To the best of our knowledge CCL21 is the first non-canonical NFκB2 target identified in tubular cells. Since TNF did not promote a similar upregulation of CCL21 at concentrations that clearly upregulate MCP-1, Il-6 and RANTES [9], our findings identify renal actions of TWEAK that are non-redundant to those of TNF. This emphasizes the importance of unraveling the role of TWEAK in renal disease. The in vivo observation of increased CCL21 during AKI as well as the modulation by TWEAK antagonism of nuclear translocation of components of the non-canonical NFκB pathway, CCL21 expression and T cell infiltration suggests that this might be a clinically relevant observation. An essential role for CCL21 in the recruitment of effector T cells to peripheral tissues was established in a model or airway challenge [33]. Interestingly, while microarray analysis did not differentially cluster the transcriptomic responses to TWEAK and TNF, it did disclose upregulation of CCL19, another NFκB2-regulated cytokine, only by TWEAK.

There is scarce prior information on the non-canonical NFκB pathway in kidney injury. Both p52 and RelB were part of NFκB DNA-binding complexes found in the kidney in mice with unilateral ureteral obstruction [42]. Reperfusion injury is associated with NIK phosphorylation [43]. Changes in the expression of NIK and RelB, including increased cytosolic NIK and RelB expression, were found in kidneys from experimental diabetic nephropathy animals [44]. However, the factors activating this pathway were not characterized, and no downstream renal targets had been identified in these studies.

In summary, we have described TWEAK as an activator of the non-canonical NFκB pathway in tubular cells, and identified one of its target genes in these cells, the chemokine CCL21. To our knowledge this is the first time that stimuli modulating non-canonical NFκB activation in tubular cells have been explored, and the first time that TWEAK has been linked to CCL21 regulation via this pathway. Activation of the non-canonical pathway may contribute to the deleterious effect of TWEAK in AKI, as anti-TWEAK antibodies decrease renal inflammation and improve renal function [9]. This information may be relevant for developing new therapeutic approaches to kidney disease.
